# The Sesquiterpene Lactone Dehydroleucodine Triggers Senescence and Apoptosis in Association with Accumulation of DNA Damage Markers

**DOI:** 10.1371/journal.pone.0053168

**Published:** 2013-01-14

**Authors:** Valeria V. Costantino, Sabrina F. Mansilla, Juliana Speroni, Celina Amaya, Darío Cuello-Carrión, Daniel R. Ciocca, Horacio A. Priestap, Manuel A. Barbieri, Vanesa Gottifredi, Luis A. Lopez

**Affiliations:** 1 Laboratory of Cell Cycle and Cytoskeleton, Instituto de Histología y Embriología Dr. M. H. Burgos (IHEM), Facultad de Ciencias Medicas, Universidad Nacional de Cuyo, Mendoza, Argentina; 2 Instituto de Medicina y Biología Experimental de Cuyo IMBECU-CONICET, Mendoza, Argentina; 3 Cell Cycle and Genomic Stability Laboratory, Fundación Instituto Leloir-CONICET, Buenos Aires, Argentina; 4 Department of Biological Sciences, Florida International University, Miami, Florida, United States of America; 5 Fairchild Tropical Botanic Garden, Coral Gables, Florida, United States of America; University of Hawaii Cancer Center, United States of America

## Abstract

Sesquiterpene lactones (SLs) are plant-derived compounds that display anti-cancer effects. Some SLs derivatives have a marked killing effect on cancer cells and have therefore reached clinical trials. Little is known regarding the mechanism of action of SLs. We studied the responses of human cancer cells exposed to various concentrations of dehydroleucodine (DhL), a SL of the guaianolide group isolated and purified from *Artemisia douglasiana* (Besser), a medicinal herb that is commonly used in Argentina. We demonstrate for the first time that treatment of cancer cells with DhL, promotes the accumulation of DNA damage markers such as phosphorylation of ATM and focal organization of γH2AX and 53BP1. This accumulation triggers cell senescence or apoptosis depending on the concentration of the DhL delivered to cells. Transient DhL treatment also induces marked accumulation of senescent cells. Our findings help elucidate the mechanism whereby DhL triggers cell cycle arrest and cell death and provide a basis for further exploration of the effects of DhL in *in vivo* cancer treatment models.

## Introduction

Sesquiterpene lactones (SLs) are a large and structurally diverse group of plant metabolites [1] many members of which display anti-tumor effects [2], [3]. The SLs of the guaianolide group are of particular interesting as anti-tumor agents because each chemical substitution to the guaianolide skeleton confers a particular biological activity to the resulting compound [2].

Although our knowledge of the mechanism of action of SLs in general is limited, some of them have reached clinical trials because of an ability to selectively trigger cell death in cancer cells while sparing normal cells [4]–[7]. The precise basis of this selective effect is still unclear; however, many studies have demonstrated an association between the anti-tumor effect of SLs and anti-inflammatory responses [8]–[11]. There is recent evidence of an intracellular cell killing effect triggered by SLs. The disruption of a calcium pump in the endoplasmic reticulum [12], [13], increased generation in iron-dependent free radicals [14], [15], control of nuclear factor κB (NFκB), activation of the tumor suppressor p53 [8], [16], [17], alteration of the epigenetic code [18], [19], and DNA alkylation [20], have all been reported as target intracellular events altered by SL treatment that could account for the anti-tumorigenic effect of SLs [2]. Collectively, the above findings indicate a potential multifactorial effect of SLs in cancer cells.

We study one particular lactone, dehydroleucodine (DhL), a SL of the guaianolide group that consists of an alpha-methylene butyrogamma-lactone ring connected to a seven-membered ring that is fused to an exocyclic alpha, beta-unsaturated cyclopentenone ring (structure: Fig. S1). The crystal structure of DhL was recently resolved [21]. DhL can be isolated and purified at concentrations >1% from the above-ground parts of *Artemisia douglasiana* (Besser), a widespread and easily obtained medicinal herb that is commonly used in Argentina [22]. We have shown previously that DhL has an antiproliferative effect on plant cells, rat cells [23], [24], and mouse B16 melanoma cells, but not on normal murine Melan-A melanocytes [25]. We have also found that DhL inhibits the re-initiation of meiosis in amphibian oocytes [26], [27]. We therefore suspected that DhL might have anti-tumor effects similar to those reported previously for other SLs of the guaianolide group. We also thought that DhL could affect at least certain aspects of the DNA damage response (DDR). Genotoxins that are used for cancer treatment usually affect cellular proliferation by increasing replication stress [28]. Alterations in the coordinated replication process typically result in the accumulation of stalled, asymmetric, or broken replication forks [29]. The defective activation of pathways that repair DNA lesions generally triggers cell death programs (*e.g.*, apoptosis), permanent cell cycle withdrawal, or senescence [30].

In the present study, we confirmed the antiproliferative effect of DhL in human cancer cells. Our analysis of the accumulation of DNA damage markers revealed a striking correlation between the extent of DNA damage and the activation of senescence and apoptosis programs, which were selectively stimulated by lower and higher DhL concentrations, respectively. Clonogenic assays revealed the very effective depletion of proliferating cells by DhL-induced apoptotic and senescence programs. Further analysis of the novel role of DhL in cellular senescence showed that the antiproliferative process was associated with a delay of the progression through the G2 phase that preceded an arrest in the following G1 phase. This phenomenon was accompanied by reduced cyclin B1 levels and higher p53 levels, suggesting that p53 has the ability to promote cell cycle withdrawal. Transient DhL treatment (8 h) was equally effective as continuous DhL treatment in terms of the numbers of cells that displayed premature senescence. Collectively, our findings indicate that DhL activates different antiproliferative programs depending on the time frame and on the concentration delivered to cells.

## Materials and Methods

### Reagents

Dehydroleucodine (DhL) (structure: Fig. S1) at 93% purity was obtained as described in our previous study [21]. 4′, 6-diamidino-2-phenylindole (DAPI) was from Sigma-Aldrich (St. Louis, MO, USA) and peroxidase-labeled streptavidin was from Dako Denmark S/A (Glostrup, Denmark). Antibodies against cyclin B1, p21, p53 and anti-53BP1 were from Santa Cruz Biotechnology (Santa Cruz, CA, USA). Antibodies against beta-actin and biotin-conjugated mouse monoclonal anti-digoxigenin were from Sigma-Aldrich. Antibodies against phospho-Histone H2AX, phospho-Histone H3 and phospho ATM were from Millipore, (California), anti-phospho Chk1 wasfrom Cell Signaling Technology, anti p53 (1801 and DO1) were gifts from Carol Prives-Columbia University New York and anti p21 was from Santa Cruz Biotechnology. Biotinylated anti-mouse antibody was from Dako Denmark S/A. Cy3-conjugated anti-mouse secondary antibody was from Jackson ImmunoResearch Inc. (West Grove, PA, USA) and Alexa 488-conjugated anti-rabbit secondary antibody was from Invitrogen/Life Technologies Corp. (Carlsbad, CA, USA).

### Cell Lines

Cell lines HeLa S3 (from human cervix carcinoma), MCF-7 (from human breast adenocarcinoma), WI-38 (from human embryonic lung fibroblast) and WI-38 VA (from human embryonic lung SV 40 virus-transformed derivate of the WI-38 cell line) were purchased from ATCC (Manassas, VA, USA) and cultured for less than 6 months. HCT116 p53+/+ (clone 40.16) and HCT116 p53−/− (clone 379.2) cells (from human rectal carcinoma) [31] were generously donated by Dr. B. Vogelstein. These cells were received from the Genetics Resource Core Facility of the Johns Hopkins University School of Medicine (Baltimore, MA, USA) in 2000. One vial of each sample was amplified for 2 passages and frozen by Vanesa Gottifredi at Columbia University (Manhattan, NY, USA). One of the vials providing from Columbia University was thawed for use in the present study in 2010. The number of passages did not exceed the number of 20 or less upon the resuscitation in the year 2010. p53 levels were checked following each recovery. Cells were grown in Dulbecco’s modified Eagle’s medium (D-MEM/F12, Gibco BRL, Gaithersburg, MD, USA) supplemented with 10% fetal bovine serum (FBS), 50 U/ml penicillin, and 50 µg/ml streptomycin in a humidified incubator with 5% CO_2_ at 37°C. The cells were harvested after reaching 70–80% confluence and were plated for either subsequent passage or treatments.

### Cell Synchronization and Treatments

HeLa cells were synchronized at the G1/S phases by a double thymidine block as described by Knehr et al. [32]. Briefly, HeLa cells (2×10^5^) were seeded in 6-well plates, treated with 4 mM thymidine for 16 h, and released by treatment with regular medium for 8 h. A second, synchronized arrest at the G1/S phases was induced by further treatment of cells with 2 mM thymidine for 14 h (these cells are referred to hereafter as “synchronized cells”). In parallel experiments, HeLa, MCF-7, WI-38, WI-38 VA, HCT116 p53+/+, and HCT116 p53−/− cells (2×10^5^) were seeded in 6-well plates in D-MEM/F12 medium with 10% FBS for 12 h (these cells are referred to hereafter as “unsynchronized cells”). Both synchronized and unsynchronized cells were released by placement in fresh medium (D-MEM/F12 with 10% FBS) containing DMSO or DhL (defined as time 0) and cultured for various durations. DMSO was used as a vehicle control in all experiments.

### Cell Proliferation Assays

Synchronized or unsynchronized cells (2×10^5^) were treated with various concentrations (0–30 µM) of DhL for 72 or 96 h. To determine cell numbers, cells were trypsinized, suspended in regular medium, and counted with a Neubauer chamber. For MTT [3-(4,5-dimethylthiazol-2-yl)-2,5-diphenyltetrazolium bromide] assay, cells were washed with PBS and 2 ml serum-free culture medium containing 1 mg/ml MTT (Sigma-Aldrich) was added to each well. The medium was discarded after 4 h, DMSO was added to dissolve MTT-derived formazan, and formazan was quantified by the measurement of absorbance at wavelength 550 nm as described previously [33].

### Apoptosis Assays

Apoptosis was assayed by TUNEL and by Annexin V staining. For Tunel assay, it was used the ApopTag Plus *in situ* detection kit (Oncor, Gaithersburg, MD, USA) as described previously [34]. Briefly, cells cultured on coverslips were treated with various concentrations (0–30 µM) of DhL for 24 or 48 h, fixed with 4% paraformaldehyde in PBS for 30 min and post-fixed with ethanol/acetic acid (2∶1) for 5 min at −20°C. Endogenous peroxidase activity was quenched by treatment with 3% H_2_O_2_ in PBS for 5 min. The coverslips were treated with 0.01 M citrate buffer (pH 3) to boiling in a microwave oven for 30 min. Nucleotides were labeled according to the manufacturer’s instructions. The coverslips were blocked with 10% bovine serum albumin (30 min at 20°C), incubated overnight with biotin-conjugated mouse monoclonal anti-digoxigenin antibody at 4°C, washed, incubated with biotinylated anti-mouse antibody for 45 min at room temperature, washed again, incubated with peroxidase-labeled streptavidin for 45 min at room temperature, washed briefly with PBS, and incubated with 0.5 mg/ml 3,3′ diaminobenzidine tetrahydrochloride/H_2_O_2_ for 10 min. The slides were lightly counterstained with hematoxylin to reveal nuclei, examined and photographed with a Nikon Eclipse E200 microscope (Nikon Corp., Tokyo, Japan). The apoptotic index was calculated as the percentage of positive nuclei based on an average of 100 cells in each experimental group in three independent experiments and expressed as % apoptotic cells ± SEM.

For the assessment of early stage apoptosis, cells were stained with Annexin V [35] using the Annexin V-FITC fluorescence detection kit (BD Biosciences San Jose, CA, USA) according to the manufacturer’s instructions. Briefly, cells cultured on coverslips were treated with various concentrations (0–30 µM) of DhL for 24, 48 or 72 h, washed with PBS twice and then once with Annexin V Binding Buffer. The cells were stained with Annexin V-FITC diluted 1∶10 in Annexin V Binding Buffer for 15 min at RT and fixed with 2% paraformaldehyde. The slides were examined and photographed with a Nikon Eclipse TE 2000 U motorized inverted microscope (Nikon Corp., Tokyo, Japan). The apoptotic index was calculated as the percentage of cells stained positive for Annexin V. 100 cells were counted in each experimental group in three independent experiments and expressed as % apoptotic cells ± SEM.

### Immunostaining and Microscopic Analysis

Cells were plated on 10-mm diameter coverslips, treated with DhL after 24 h, fixed in 4% paraformaldehyde/sucrose for 20 min at room temperature, incubated with 0.1% Triton X-100 for 15 min and blocked overnight in PBS/2% donkey serum (Sigma-Aldrich). The coverslips were incubated for 1 h with primary antibodies as below, washed and incubated for 1 h with secondary antibodies conjugated to fluorophores. Whole nuclei were visualized using DAPI. The primary antibodies used were anti-γH2AX, anti-pH3 and anti-53BP1. The secondary antibodies used were Cy3-conjugated anti-mouse and Alexa 488-conjugated anti-rabbit. Images were obtained with a Zeiss Axioplan confocal microscope or a Zeiss Axio Imager A2 (Carl Zeiss, Oberkochen, Germany). 200 cells were counted for each time point in three independent experiments.

### Clonogenic Survival Assay

Synchronized HeLa cells (2×10^5^) were treated with various concentrations (0–30 µM) of DhL for 48 h, trypsinized, suspended in regular medium and counted. Viable cells (500/well) were seeded in 6-well plates and cultured for 10 days until colonies were large enough (≥50 cells) to be clearly discerned. The medium was then removed, and the cells were washed with PBS and fixed with 100% methanol for 30 min. The fixed colonies were stained with a filtered solution of 0.5% (w/v) crystal violet (Sigma-Aldrich) for 10 min. The staining solution was removed, and the colonies were scored manually. The clonogenic survival was expressed as a percentage relative to the number of colonies formed in DMSO-treated cells (control). Assays were performed in triplicate for each DhL concentration and time period.

### Cell Cycle Analysis

Cell DNA content was determined by flow cytometry. Following treatment, cells were washed with PBS, trypsinized, pelleted by centrifugation at 3,000 rpm for 5 min and fixed with 70% ethanol (20 min, −20°C). The fixed samples were incubated with 50 µg/ml propidium iodide and 0.1 µg/ml RNase and then analyzed on a flow cytometer (BD FASCanto II, Becton Dickinson, Franklin Lakes, NJ, USA). A total of 1×10^4^ events were analyzed using the WINMDI 2.8 software program (Joe Trotter, WinMDI, Scripps Institute, La Jolla, CA, USA; http://fac.-scripps.edu). Histograms were analyzed using the Cylchred program (Terry Hoy, Cylchred, Cardiff University, UK; http://cardiff.acuk).

### Live Cell Time-lapse Microscopy and Analysis

Phase-contrast time-lapse microscopy was performed on synchronized HeLa cells treated with various concentrations (0–20 µM) of DhL. Following the removal of the second thymidine block, 100 independent cells for each condition were recorded using a Nikon Eclipse TE 2000 U motorized inverted microscope (Nikon Corp., Tokyo, Japan) with an incubation chamber (37°C, 5% CO_2_) and a Hamamatsu ORCA-ER cooled CCD camera (Hamamatsu Photonics, Tokyo, Japan). Time-lapse videos were reviewed and the timing of the various phases of mitosis was determined using the Image J software program (Rasband WS, Image J. Bethesda, MD: National Institutes of Health: 28 October, 2003. http://rsbweb.nih.gov/ij/).

### Gel Electrophoresis and Western Blot Analysis

Treated cells were washed twice with ice-cold PBS, pelleted and lysed by freeze/thaw in extraction buffer (50 mM HEPES, pH 7.5, 1 mM EDTA, 150 mM NaCl, 10 mM β-glycerophosphate, 1 mM NaF, 0.1% Triton X-100, 10% glycerol, with protease inhibitors) for 30 min on ice. The samples were centrifuged at 12,000 rpm for 20 min at 4°C and supernatants were collected. Protein concentrations were determined by the Bradford method [36]. Cell extracts were separated by SDS-PAGE and transferred onto nitrocellulose membranes (BioRad Laboratories, Hercules, CA, USA). The membranes were incubated with antibodies against cyclin B1, p21, p53 and β-actin for 2 h at room temperature and overnight at 4°C with antibodies against phospho-ATM and phospho-Chk1. Subsequently, membranes were incubated with the appropriate horseradish peroxidase-conjugated secondary antibodies. A SuperSignal West Pico chemiluminescent substrate kit (Pierce/ThermoFisher Scientific Inc., Rockford, IL, USA) was used to visualize protein bands. Band densities were determined using the Image J program.

### Senescence-associated β-galactosidase Assays

Senescence-associated β-galactosidase (SA-β-Gal) activity was monitored in cell extracts and *in situ*. Soluble SA-β-Gal levels in cell extracts were determined as described by Lee *et al.* [37]. Briefly, equal numbers of cells were collected for untreated or treated conditions, washed, resuspended in extraction buffer and lysed by freeze/thaw. The samples were centrifuged at 12,000×g for 10 min, and the resulting supernatants were mixed with 2.2 µg/µl 1,4-methyl-umbelliferon-β-D-galactopyranoside (Sigma-Aldrich) in 1 mM MgCl_2_ phosphate buffer, pH 6 and incubated at 37°C for 2 h. The reaction was terminated by addition of two volumes of 1 M sodium carbonate. SA-β-Gal activity was monitored by measuring fluorescence emission at 360–448 nm with an Aminco Bowmen II Spectrophotofluorometer (American Instrument Co., Silver Spring, MD, USA). Protein concentrations were determined by the Bradford method.

*In situ* SA-β-Gal staining was performed as described by Dimri *et al.* [38]. Briefly, cells were fixed with 2% formaldehyde and 0.2% glutaraldehyde for 5 min and incubated overnight at 37°C with 1 mg/ml X-gal staining solution (5-bromo-4-chloro-3-indolyl β-D-galactoside, 5 mM K_3_Fe[CN]_6_, 5 mM K_4_Fe[CN]_6_, and 2 mM MgCl_2_ in PBS, pH 6.0). The cells were rinsed twice with PBS, washed with methanol and examined using a Nikon Eclipse E200 microscope (Nikon Corp., Tokyo, Japan).

### Senescence-associated Heterochromatin Foci (SAHF) Assay

Cells grown on chamber slides were fixed with 2% paraformaldehyde for 30 min, washed with PBS, incubated with 0.2% Triton X-100 in PBS for 1 h, washed with PBS and incubated with PBS containing 1 µg/ml DAPI at room temperature for 5 min. The number of cells in SAHF assay was determined using a confocal microscope (Olympus FV-1000; Olympus, Tokyo, Japan) with an excitation wavelength of 350 nm. A total of 100 cells were analyzed for each time point in two independent experiments.

### Statistical Analysis

The data shown are mean ± SEM from 2 or 3 independent experiments. Statistical analyses were performed using one-way ANOVA or paired two-tailed Student’s *t*-test (Prism 5 program, GraphPad Software Inc., La Jolla, CA, USA). Differences were considered significant for p values ≤0.05.

## Results

### DhL Inhibits the Proliferation of Human Cancer Cells

**Figure 1:**
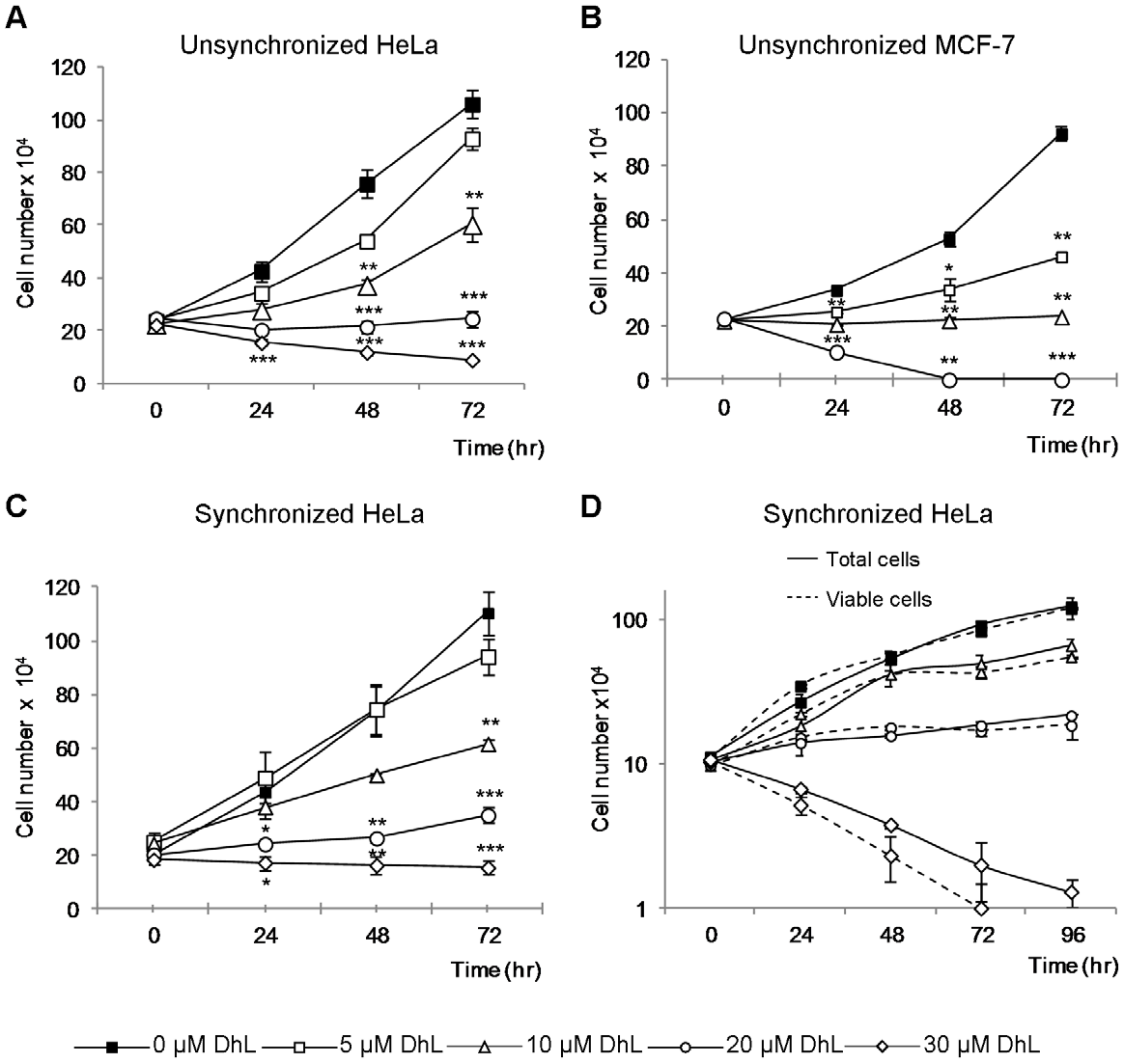
DhL treatment inhibits cell proliferation in a dose-dependent manner. Unsynchronized HeLa (A) and MCF-7 cells (B) and synchronized HeLa (C and D) cells were treated with 0, 5, 10, 20, or 30 µM DhL for 72 or 96 h and counted every 24 h. The total number of cells counted each 24 h (Total cells) were compared with the number of viable cells (Viable cells) (D). Data are expressed as the mean ± SEM of 3 independent experiments. (A), (B) and (C) * p≤0.05, ** p≤0.01, *** p≤0.001 vs. control group (0 µM DhL). (D) * p≤0.05, ** p≤0.01, *** p≤0.001 total cells vs. viable cells.

To investigate the effects of DhL on human cancer cells, we first examined its effects on cell proliferation. HeLa and MCF-7 cells were treated with various concentrations of DhL for 72 h, and the effect on cell growth was evaluated by cell counting. The half maximal inhibitory concentration (IC50) of DhL at 72 h culture for HeLa cells was 10 µM (Fig. 1A). The cell number further decreased to 80% when 20 µM DhL was used. The IC50 of DhL at 72 h culture for MCF-7 cells was 5 µM (Fig. 1B). Similar effects were observed when DhL was added to synchronized HeLa cells and cell proliferation was assessed by cell count (Fig. 1C) or by MTT assay (Fig. 1D). The estimation of the number of total cells and the number of viable cells each 24 h indicated that during 96 h of DhL treatments, only 30 µM DhL affected the viability of the cells (Fig. 1D).

In order to asses if DhL inhibition of proliferation is selective of cancer cells, we performed cell counting experiments on normal WI-38 lung fibroblasts and transformed WI-38 VA cells. The IC50 of DhL at 72 h culture was 4 µM for WI-38 cells and 14 µM for WI-38 VA cells (Fig. S1B) showing that WI-38 VA cells are more sensitive to DhL treatment. While a cell line-dependent drug effect cannot be ruled out, these findings indicated that the proliferation of several tumor cell lines is inhibited by DhL in a dose-dependent manner.

### DhL does not Induce Massive Apoptosis at 20 µM Concentration

**Figure 2:**
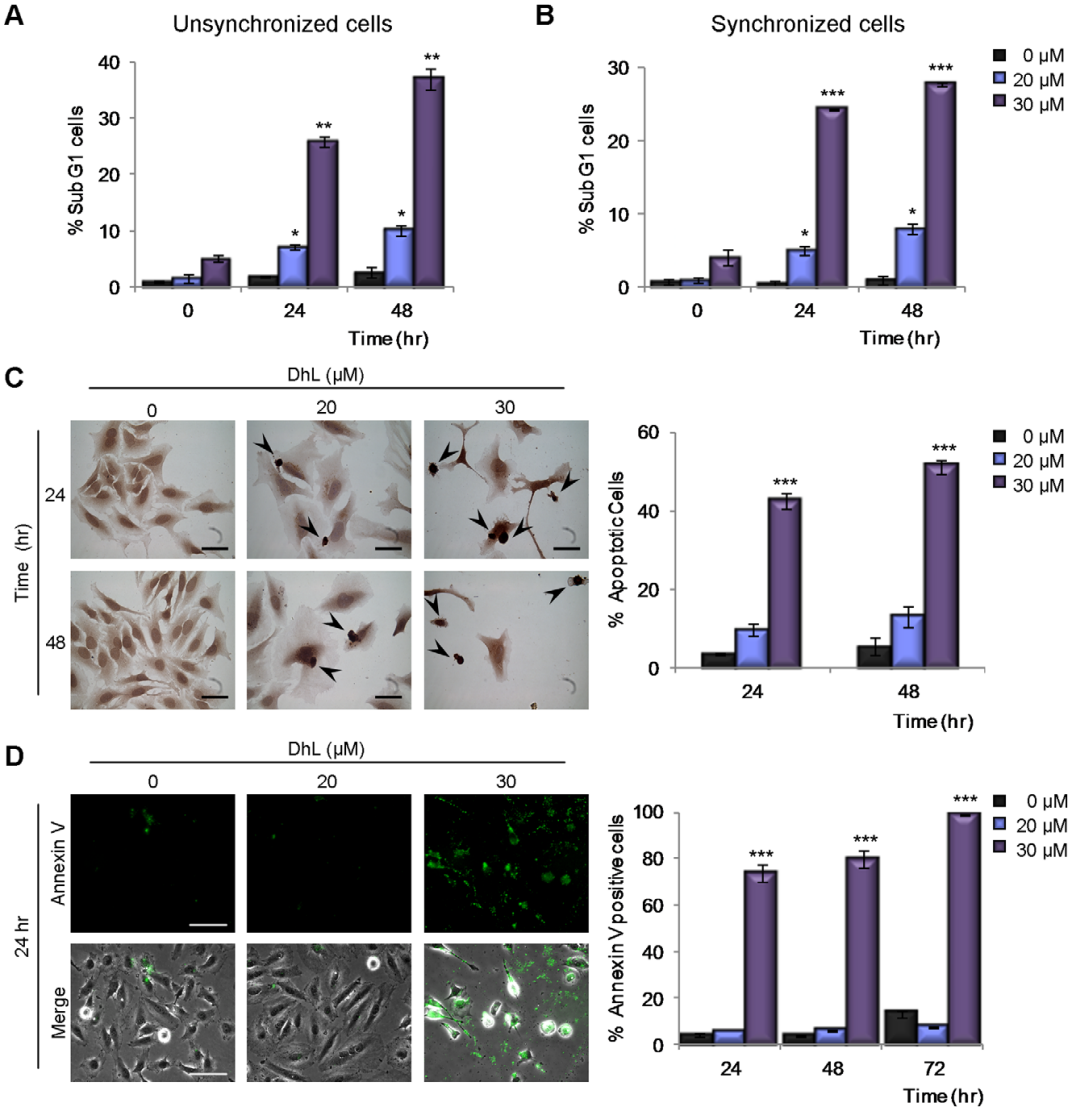
Treatment with 30 µM DhL induces apoptosis in HeLa cells. Unsynchronized (A) and synchronized (B) HeLa cells were treated with 0, 20, or 30 µM DhL for 24 or 48 h. DNA content was assessed by flow cytometry (see Fig. S2, DNA distributions). The percentages of unsynchronized and synchronized cells in the sub-G1 phase are shown. (C) Synchronized HeLa cells were treated with 0, 20, or 30 µM DhL for 24 or 48 h. Apoptotic cells were assessed by TUNEL assay. Left: representative panels with apoptotic cells indicated by arrowheads. Bar: 50 µm. Right: percentages of apoptotic cells at 24 and 48 h. (D) Unsynchronized HeLa cells were treated as in (C) and subjected to Annexin V assay. Left: representative panels with apoptotic cells stained with Annexin V (bright cells) at 24 h. Representative fields of Annexin V positive cells for 48 and 72 h treatment are shown in Fig. S2 C. Bar: 50 µm. Right: percentages of apoptotic cells at 24, 48 and 72 h. Data represent mean ± SEM of 3 independent experiments. * p≤0.05, ** p≤0.01, *** p≤0.001 vs. control group (0 µM DhL).

The reduction in cell number upon DhL addition could result from cell death, cell cycle arrest or both. We initially evaluated the extent of cell death induced by DhL treatment. Because high doses of DhL clearly reduced cell proliferation in both cell lines and in both proliferation assays (Fig. 1), we analyzed the distribution of HeLa cells at various cell cycle stages following treatment with 20 and 30 µM DhL. Signals of cell death in terms of the presence of a sub-G1 phase increased only weakly when cells were incubated with 20 µM DhL. In striking contrast, a massive increase in the sub-G1 phase population was observed when cells were treated with 30 µM DhL (Figs. 2A and S2A). Similar results were obtained when HeLa cells that were synchronized in the G1/S phase were released in growth medium containing DhL (Figs. 2B and S2B). These findings indicate that HeLa cell death was specifically upregulated when the DhL dose was 30 µM.

To determine whether the cell death induced by DhL was apoptotic, we used the specific TUNEL assay. A DhL concentration of 20 µM induced only a slight increase in the TUNEL signal (*i.e.*, the percentage of cells with TUNEL-positive DNA) following 24 or 48 h of treatment, whereas a DhL concentration of 30 µM caused a considerable increase in the TUNEL signal (Fig. 2C). Similar results were obtained when scoring for Anexin V positive cells (Fig. 2D and S2C). These findings indicate that a high DhL concentration promoted the apoptotic death of HeLa cells whereas a lower concentration reduces cell number without causing an increase in death markers.

**Figure 3:**
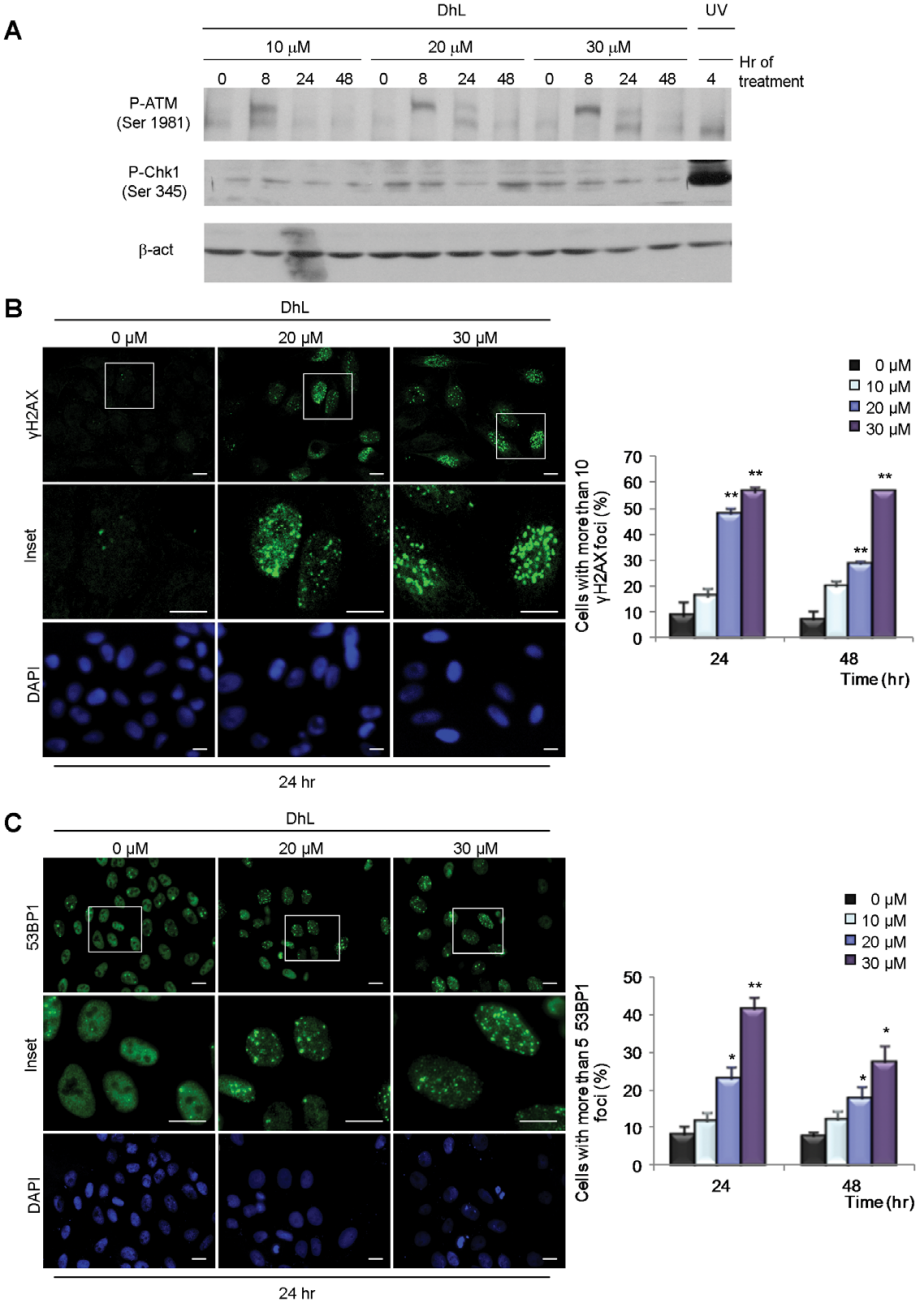
DhL treatment causes the accumulation of DNA damage markers. Unsynchronized HeLa cells were treated with 0, 20, or 30 µM DhL and lysed or fixed at the indicated times. (A) Immunoblot analysis of phospho-ATM (p-ATM) and phospho-Chk1 (p-Chk1). Representative assay of 3 independent experiments. β-actin was employed as a loading control. Cells were stained with DAPI to visualize the nuclei and treated with specific antibodies for γH2AX (B) and 53BP1 (C). Left: representative fields from 24 h treatment. Insets: magnification of the areas indicated by boxes in the top row. Representative fields for 48 h treatment are shown in Fig. S3. Right: quantification of the number of cells with more than 10 γH2AX foci and more than 5 53BP1 foci. At least 200 nuclei were scored for each sample. Bar: 10 µm. Data represent mean ± SEM of 3 independent experiments. * p≤0.05, ** p≤0.01 vs. control group (0 µM DhL).

Since cell death and apoptosis can result from the activation of checkpoint kinases involved in the activation of the DDR response we analyzed the effect on DhL in the phosphorylation/activation of checkpoint kinases ATM and Chk1. We observed that ATM, but not Chk1, was phosphorylated by treatment with all concentrations of DhL reaching maximal phosphorylation at 8 h of treatment (Figs. 3A and S3A). We then analyzed the levels of histone H2AX phosphorylation, which is associated with the activation of checkpoint kinases in the chromatin microenvironment surrounding damaged DNA [39]. DhL concentrations of 20 and 30 µM (but not 10 µM) induced the accumulation of phosphorylated H2AX (γH2AX) foci at 24 h (Fig. 3B), indicating that both the antiproliferative and the apoptotic effect of DhL were associated with the accumulation of damaged DNA. A reduction in the number of cells with γH2AX foci was observed following 48 h treatment with 20 µM DhL (Figs. 3B and S3B). In contrast, no reduction in this parameter was observed following 48 h treatment with 30 µM DhL (Figs. 3B and S3B), suggesting that increased amounts of irreversible DNA damage may be associated with the increased apoptosis triggered by the higher (30 µM) DhL concentration.

Replication-associated irreversible errors have been linked with the accumulation of double strand breaks (DSBs). ATM activation has also been linked to DSBs accumulation. DSBs are the most deleterious type of DNA lesion and may have severe consequences for cell survival if left unrepaired [40]. We analyzed the subnuclear organization of 53BP1, a well-established DSB marker [41]. DhL concentrations of both 20 and 30 µM increased the number of 53BP1-positive HeLa cells at 24 and 48 h of treatment (Figs. 3C and S3C), with the 30 µM concentration being the stronger inducer of 53BP1 focal accumulation (Fig. 3C). Collectively, these findings indicate that DhL might cause accumulation of DNA lesions that trigger the activation of ATM and downstream markers of DDR response.

### DhL Causes a Transient Arrest in the G2/M Phase Followed by Cell Accumulation in the G1 Phase of the Following Cycle

**Figure 4:**
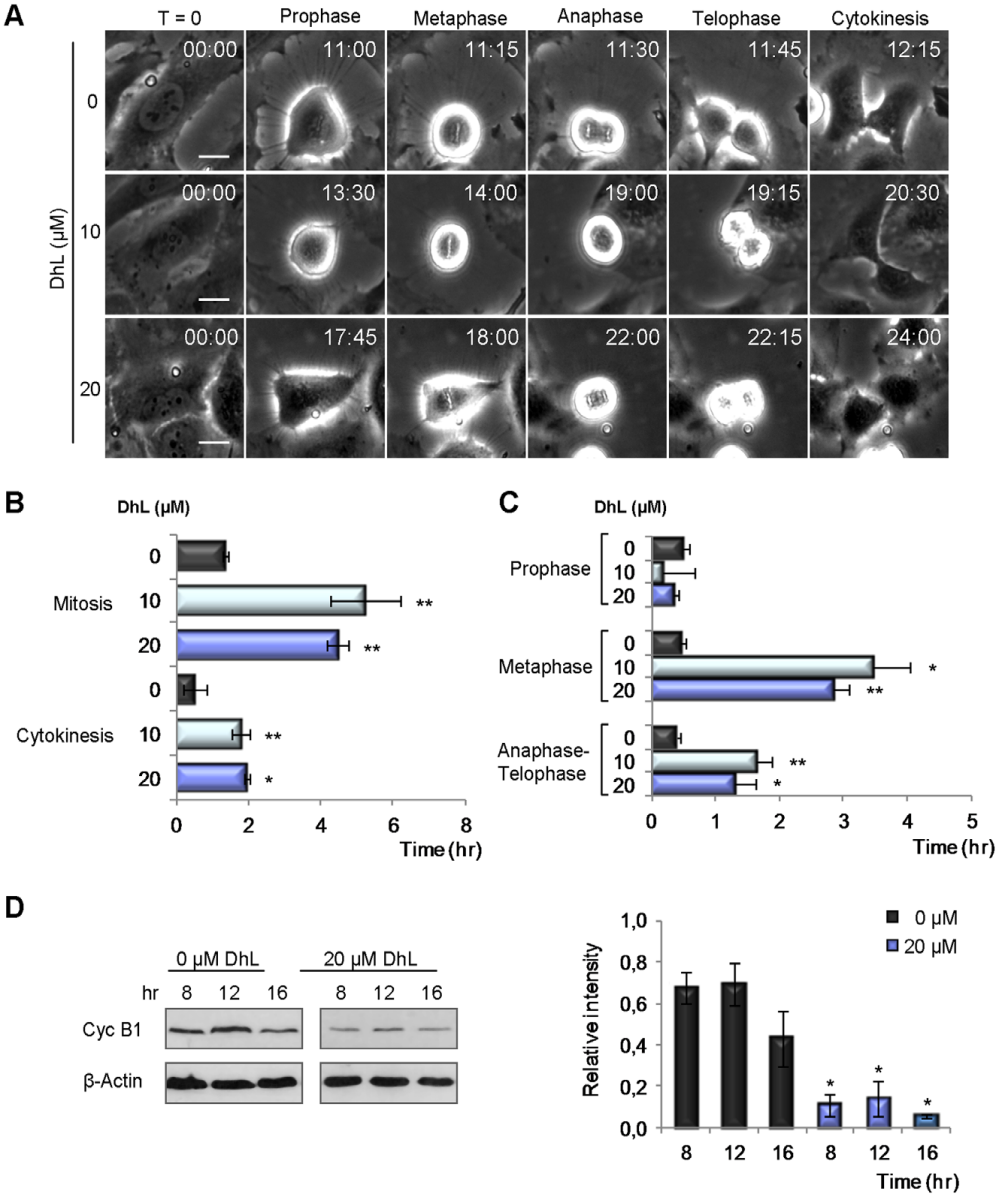
DhL delays mitosis entry extends the permanence of mitosis, and downregulates cyclin B1. Synchronized HeLa cells were treated with 0, 10, or 20 µM DhL and analyzed by phase contrast in live time-lapse microscopy from 0 to 32 h treatment; images were acquired every 15 min using a Nikon Eclipse TE 2000-U microscope. (A) Representative images of cells at the beginning of treatment (T = 0), prophase, metaphase, anaphase, telophase, and cytokinesis. Upper right of each panel: average time (h:min) to reach each phase. Bar: 20 µm. The average times of mitosis and cytokinesis (B) and of the phases of mitosis (C) were calculated by analyzing 4 movies following 200 cells for each treatment. (D) Immunoblot analysis of cyclin B1. Left: immunoblot representative of 3 independent experiments. β-actin was employed as a loading control. Right: mean intensity ± SEM obtained from densitometric analysis of 3 independent experiments. * p≤0.05, ** p≤0.01 vs. control group (0 µM DhL).

In view of the observation that 20 µM DhL caused the accumulation of DNA damage, we examined the mechanism whereby this DhL concentration caused an apoptosis-independent reduction in cell number. To determine initially whether DhL caused transient or permanent delays in any phase of the cell cycle, we examined synchronized HeLa cells by live time-lapse microscopy and compared the kinetics of mitosis entrance and exit in control (mock-treated) and DhL-treated cells. There was a significant increase in the timing of mitosis entrance in DhL-treated cells. Control cells entered mitosis at 11.7±0.5 h, whereas cells treated with 10 or 20 µM DhL entered mitosis at 16.0±1.7 h or 16.2±0.5 h, respectively (Fig. 4A). The time that cells spent in mitosis was also altered by DhL treatment. Control cells spent an average of 1.9±0.1 h in mitosis, whereas DhL-treated cells remained in mitosis an average of 4.8 h longer (Fig. 4B). This delayed transition through mitosis was observed mainly at metaphase-telophase (Fig. 4C). The treated cells spent 1.4 h longer in cytokinesis that did control cells (Fig. 4B). In view of the striking delay in the G2/M phase caused by DhL treatment, we evaluated the levels of cyclin B1, a key factor that positively modulates the G2/M transition [42]. The concentration of cyclin B1 was significantly lower in treated cells, consistently with the slow progression through the G2/M phase (Fig. 4D). This finding provided molecular evidence for the inhibitory role of DhL in G2/M progression.

**Figure 5:**
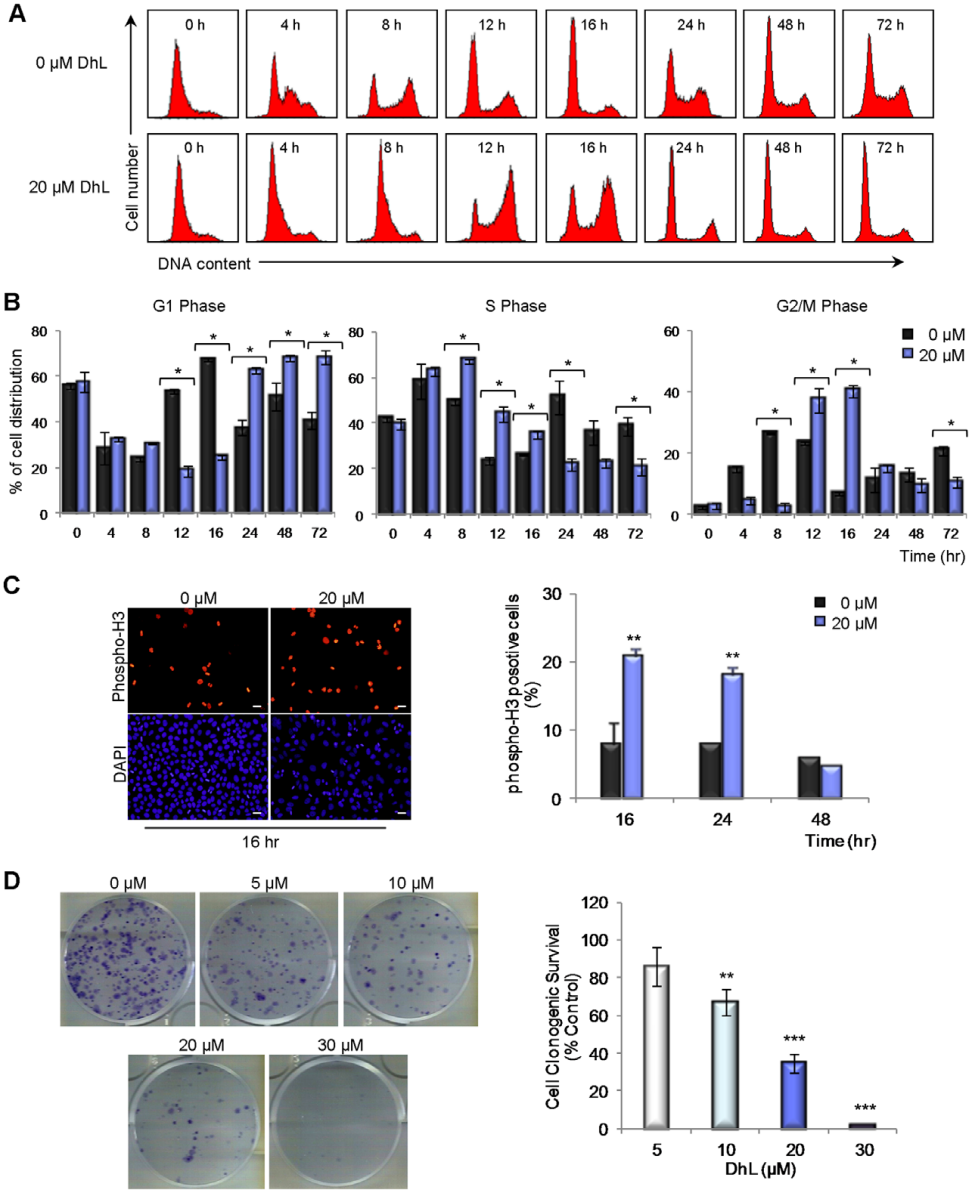
20 µM DhL inhibits cell growth by inducing transient arrest in the G2/M phase and permanent accumulation in the G1 phase. (A) Synchronized HeLa cells were treated with 0–20 µM DhL for 72 h and subjected to DNA flow cytometry at indicated times. Representative DNA distributions from one experiment are shown. (B) Percentage of cells in G1 (left panel), S (middle panel), and G2/M (right panel) phases were determined using the WinMDI 2.9 program. (C) Left: unsynchronized HeLa cells were treated with 0–20 µM DhL and stained with DAPI (to visualize nuclei) and antibodies specific to phospho-H3 at the indicated times. Right: percentage of phospho-H3 positive cells. Bar: 10 µm. (D) Cells were treated with the indicated concentrations of DhL for 48 h, counted, and replated after treatment. Cells that had the ability to form colonies were scored based on clonogenic survival assay 10 days post-treatment. Left: fixed and stained colonies from each treatment representative of 3 independent experiments. Right: number of colonies counted expressed as a percentage of the control (defined as 100%). Data represent mean ± SEM of 3 independent experiments. * p≤0.05, ** p≤0.01, *** p≤0.001 vs. control group (0 µM DhL).

We examined the possibility that defects associated with DhL treatment had an irreversible impact on the cell cycle. Synchronized HeLa cells were released in culture media containing either DMSO or 20 µM DhL, and the cell cycle distribution at various time points was evaluated by flow cytometric analysis. A clear delay in the transition through the first S and G2/M phase was observed within the first 12 h in DhL-treated cells (Fig. 5A, B). At 24, 48 and 72 h, while DhL-treated cells accumulated in the following G1 phase, control cells were transitioning through not only the G1 phase but also the following S phase (Fig. 5A, B). These findings indicated that cell cycle progression was delayed in the treated cells.

We analyzed the phosphorylation of histone H3 in asynchronous populations of DhL-treated HeLa cells and found an increase in the number of cells that scored positive for this M-phase marker (Fig. 5C, 16 h time point). This finding suggested that cells undergoing the first G2/M phase transition following DhL treatment experienced longer, transient stops in the G2 and M phases that control cell populations (see decay at later time points in Fig. 5C).

The alteration of transitions through the cell cycle phases and the strong accumulation of DDR markers caused by 20 µM DhL suggested that this concentration triggered a prolonged cell cycle arrest at the G1 phase. We considered the possibility that, despite the lack of apoptotic response to treatment with 20 µM DhL, a permanent arrest in G1 phase may have reduced the colony-forming ability of HeLa cells. We observed a reduction in colony formation in cells that were replated following treatment with 30 µM DhL. Treatment with 20 µM DhL significantly reduced the number of colonies (Fig. 5D) despite its modest effect on apoptosis (Fig. 2). Collectively, these findings suggest that treatment with 20 µM DhL may cause permanent withdrawal from the cell cycle.

### The p53-p21 Pathway is Upregulated by DhL

**Figure 6:**
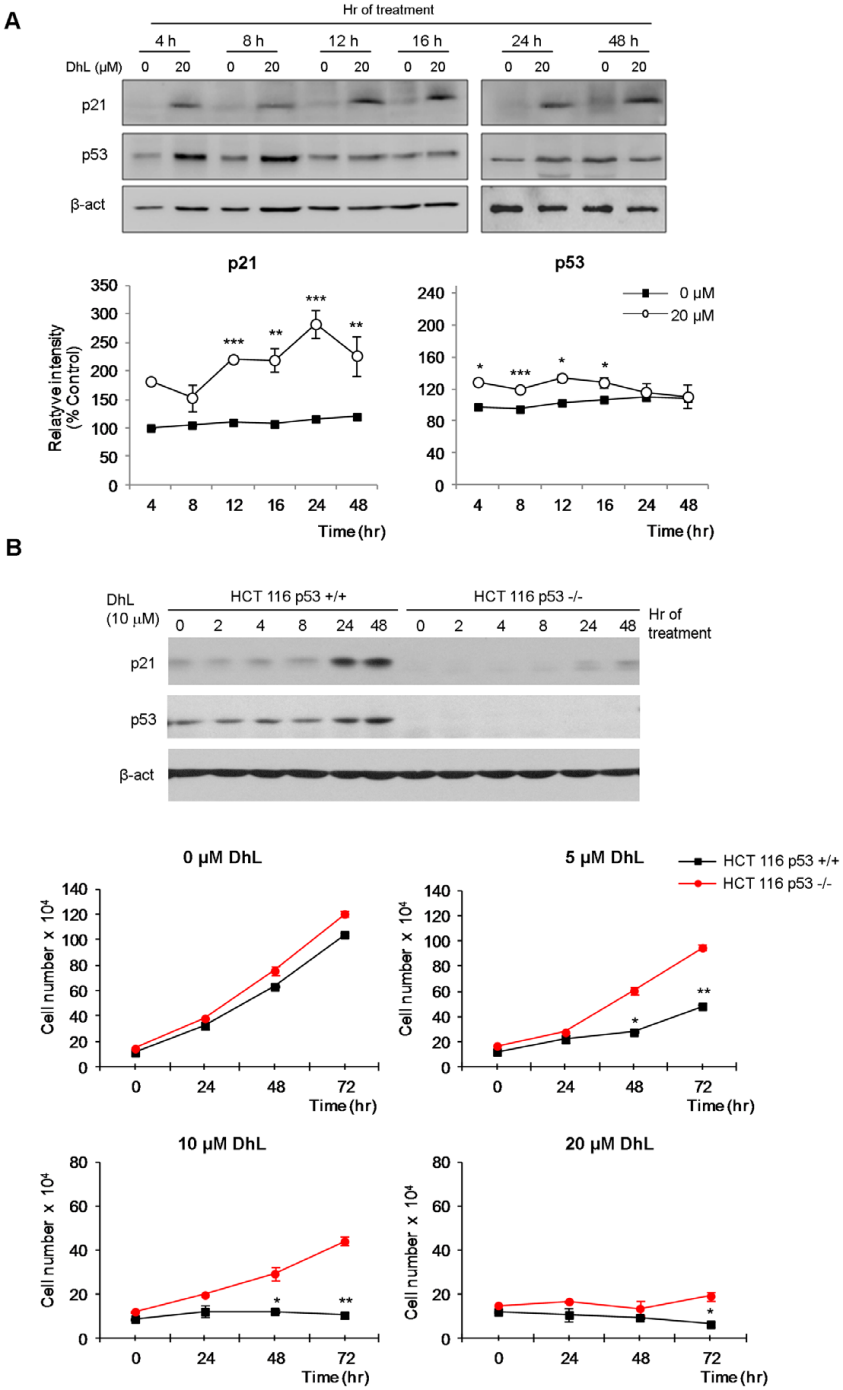
p53 sensitizes cells to the antiproliferative effect of DhL. (A) Synchronized HeLa cells treated with 0–20 µM of DhL were lysed at the indicated times. Immunoblot analyses of proteins p53 and its transcriptional target p21 are shown. Lower plots: densitometric analysis of p21 and p53 normalized to β-actin. Relative intensities of the bands are expressing percentages relative to the control (defined as 100%). The immunoblots shown are representative of 3 independent experiments with similar result. * p≤0.05, ** p≤0.01 *** p≤0.001 for the treated group (20 µM DhL) vs. control group (0 µM DhL). (B) Unsynchronized HCT116 p53+/+ and HCT116 p53−/− cells were treated with 0, 5, 10, or 20 µM DhL, lysed at the indicate times and proteins p53 and p21 were analyzed by immunoblot. β-actin was employed as a loading control (upper panel) or counted at the indicated times (lower panel). The immunoblot shown are representative of 3 independent experiments with similar result. Data represent the mean ± SEM of 3 experiments, * p≤0.05, ** p≤0.01 for HCT116 p53+/+ vs. HCT116 p53−/−.

The permanent cell cycle arrest of HeLa cells following 20 µM DhL treatment suggested that antiproliferative signals may be upregulated by DhL. Furthermore, our observations regarding the effect of DhL on γH2AX and 53BP1 accumulation indicated that targets of the ATR/ATM-Chk kinase pathway might be activated [43]. One of the most studied effector molecules of the checkpoint pathway is the tumor suppressor p53, which is a universal target of genotoxins. We therefore examined the possible alteration of p53 activity by DhL treatment. We observed increased p21 levels following DhL treatment at all times tested (Fig. 6A), consistently with our results for G1 phase accumulation (Fig. 5A, B). We also found a transient increase of p53 up to 16 h (Fig. 6A). Because the accumulation of p53 is generally associated with the activation of checkpoint signal proteins, we inferred that DNA damage caused by DhL treatment could trigger p53 accumulation and activation.

Finally, we considered the possibility that activation of the p53 signaling pathway played a role in the antiproliferative effect of DhL. We used HCT116 p53+/+ cells and their isogenic derivative HCT116 p53−/− cells [44] to test this idea. First, we evaluated the effect of DhL on the accumulation of the p53 target, p21 and we observed a much more evident accumulation of p21 in HCT116 p53+/+ which suggested that DhL cause p53 transcriptional activation (Figs. 6B upper immunoblot panels and S4A). Furthermore, DhL had a clear antiproliferative effect on both cell lines, but the effect was stronger on the HCT116 p53+/+ cells, particularly at DhL concentrations below 20 µM (Fig. 6B lower panels). In fact, MCF-7 (Fig.1) and HCT116 cells (this figure), both express wild type p53, and are more sensitive than HCT116 p53−/− or even HeLa cells to the growth suppressive effects of DhL at concentrations below 20 µM. These findings indicate that p53 partially mediates the antiproliferative effect of low concentrations of DhL.

### DhL Induces Premature Senescence

**Figure 7:**
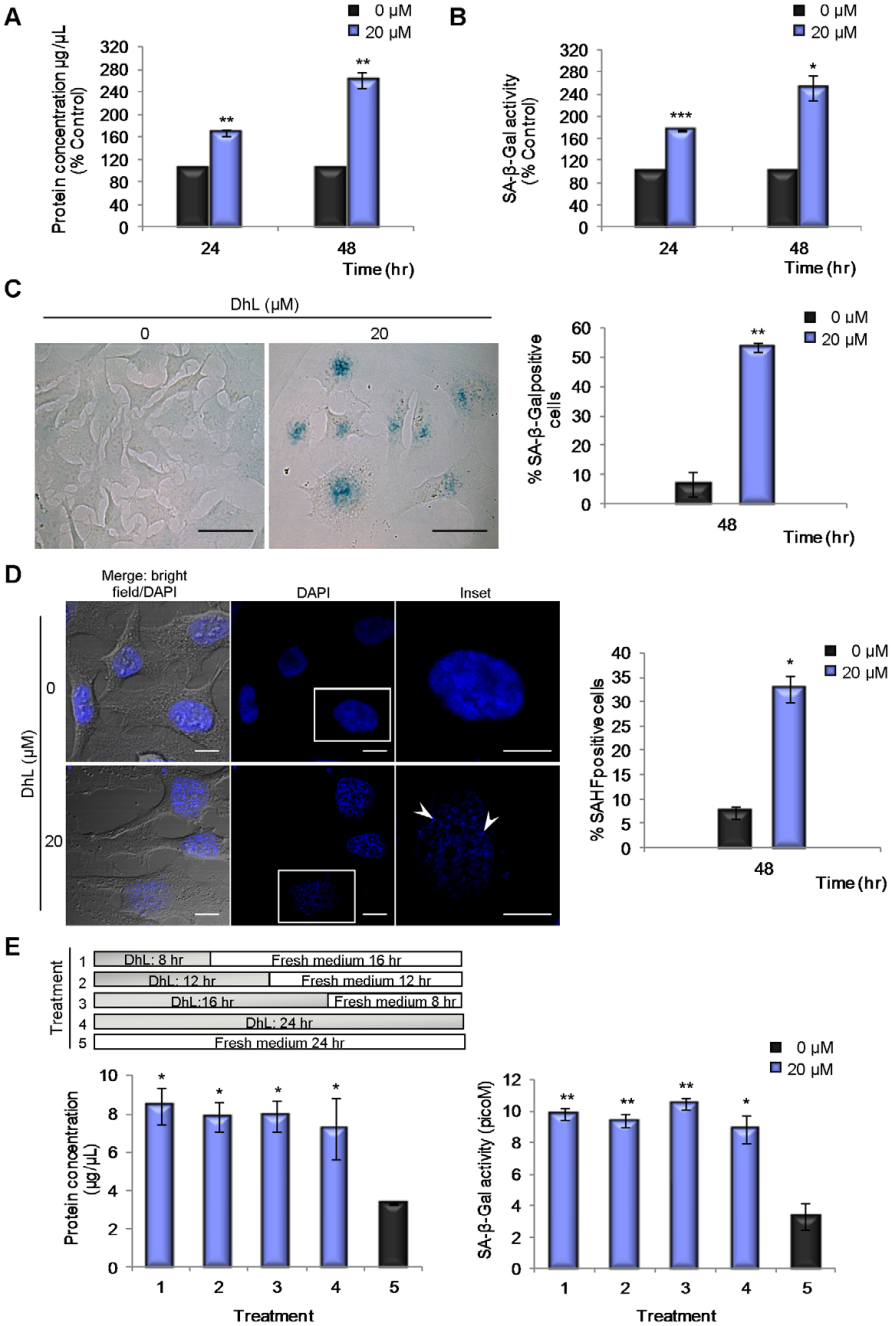
DhL induces cellular senescence in HeLa cells. Synchronized HeLa cells treated with 0–20 µM DhL for 48 h were analyzed for: (A) protein concentration and (B) SA-β-Gal activity (at pH 6) in cell extracts. Equal volumes of supernatants were assayed. C) SA-β-Gal activity at pH 6 *in situ*. Representative panels for control and treated cells stained for SA-β-Gal and examined by bright field microscopy are shown. Bar: 50 µm. Right: percentages of SA-β-Gal-positive cells. (D) Senescence-associated heterochromatin foci (SAHF) in control and treated cells stained with DAPI and examined by bright field and fluorescence microscopy (merge: bright field/DAPI) and fluorescence microscopy (DAPI). The insets are magnifications of the boxed areas in the DAPI column. Arrowheads indicate the SAHF. Bar: 10 µm. Right: percentages of SAHF-positive cells. (E) Synchronized HeLa cells treated with 20 µM DhL and then with fresh medium plus DMSO (“Fresh medium”) for the time indicated in the upper panel (treatment 1–4) or with fresh medium for 24 h (treatment 5) were analyzed for protein concentration and for SA-β-Gal activity at pH 6. The protein concentration (lower left) and SA-β-Gal activity (lower right) in cell extracts from each treatment are shown. Equal volumes of extract were assayed. Data represents the mean ± SEM of 2 experiments. * p≤0.05, ** p≤0.01, *** p≤0.001 vs. control group (0 µM).

The permanent arrest in the G1 phase mediated by p53 has been linked in most cases with DNA damage-induced senescence [44]. We considered the possibility that DhL promotes senescence in HeLa cells. Bulk protein concentration has been found to increase in senescent cells [45]. We therefore measured the protein concentration and monitored the enzymatic activity of SA-β-Gal in HeLa cell extracts at 24 and 48 h following DhL treatment. Both of these senescence parameters showed increases following treatment with 20 µM DhL (Fig. 7A, B). Consistently with this finding, we observed that DhL-treated cells were larger and flatter than control cells and showed a striking increase in the number of cells that stained positive for SA-β-Gal activity *in situ* (Fig. 7C).

Previous studies have shown that heterochromatin in senescent cells is organized into foci (SAHF) that are not observed in cycling cells [46]. We observed a marked increase of cells with SAHF following DhL treatment (Fig. 7D). Collectively, the above findings suggest that the apoptosis-free antiproliferative effect of 20 µM DhL is associated with premature senescence.

p53 transcriptional activity was linked previously to the onset of the senescence process [47]. We evaluated the contribution of p53 to the upregulation of senescence using a 10 µM concentration of DhL. This low concentration inhibited proliferation more efficiently in HCT116 p53+/+ cells than in HCT116 p53−/− cells (Fig. 6B lower panels). In senescence assays using 10 µM DhL we observed senescent cell percentages of 64% in HCT116 p53+/+ cells but only 38% in HCT116 p53−/− cells (Fig. S4B). Since we observed detectable senescence in HCT116 p53−/− cells and HeLa (cells that might have a down modulated p53 pathway), this finding supports the idea that p53 might partially contribute to the senescent effect of DhL.

### Transient Incubation with DhL Promotes Senescence in HeLa Cells

Partially in view of the potential application of DhL in the treatment of patients, we examined the degree of senescence caused by short-term exposure of HeLa cells to DhL using an experimental protocol in which senescence markers were monitored in cells treated with DhL for various durations. Following the treatment, DhL was removed by washing and the cells were incubated with fresh medium for a total of 24 h of culture (Fig. 7E, upper panel). The increase in protein concentration and SA-β-Gal accumulation for cells incubated with 20 µM DhL for 8 h followed by 16 h incubation without DhL was the same as for cells incubated continuously with DhL for 24 h (Fig. 7E). This finding suggests that DhL is a powerful antiproliferative agent even when administered for a limited duration. This effect should be further explored in mouse models of cancer treatment.

## Discussion

The results of this study show that the reduced proliferation of cancer cells treated with DhL is accompanied by increased focal organization of DNA-damage sensors (γ-H2AX and 53BP1) and increased levels and activity of p53. We therefore infer that DhL triggers at least certain aspects of the DDR response [41], which in turn activates apoptotic and senescence programs that are closely associated with the levels of DNA damage [30]. A permanent arrest of the G1 phase can be activated by not only low DhL concentrations but also transient exposure to DhL.

### The Accumulation of DNA Damage and the Cellular Response to DhL

Our findings suggest that the choice between apoptosis and senescence in DhL-treated HeLa cells is linked to the amount of DNA damage caused by DhL. Our analysis of γH2AX foci assembly showed that an apoptosis-inducing concentration of DhL caused a higher and more sustained accumulation of this marker (Fig. 3B, 48 h time point). The apoptosis induced by 30 µM DhL might be preceded by a failure of cells with high levels of damaged DNA to progress normally through the cell cycle (Fig. S2A, B FACS analysis). This cell death may in turn result in the excessive collapse of replication forks and sustained accumulation of DSBs [40]. We can infer the latter from the increase of phosphorylated ATM at 8 hours of DhL treatment and the accumulation of DSB markers (such as the 53BP1 foci assembly) that are detected at all time points following treatment with 30 µM DhL (Fig. 3C). The cell death observed following this treatment may result from a failure to correctly replicate DNA in the cells that are transitioning through the S phase. The reduced but still detectable levels of DNA damage (indicated by the analysis of phospho-ATM, γH2AX and 53BP1 foci accumulation) that result from the 20 µM DhL treatment may allow the improved handling of DNA lesions, more accurate but slower transitions through the S and G2/M phase (Figs. 4 and 5) and the final accumulation of cells in a G1 phase senescence equilibrium (Fig. 7). Collectively, our findings indicate that the accumulation of DhL-triggered DNA lesions activates apoptotic or senescence responses that greatly impair both short- and long-term proliferation potential (Figs. 1 and 5D).

### DhL Triggers Cellular Senescence and Apoptosis in a Concentration-dependent Manner

We have presented multiple lines of evidence that DhL causes apoptosis and senescence in HeLa and HCT116 cells. DhL at a concentration of 30 µM specifically increased the sub-G1 phase population (Figs. 2A, B and S2A, B), the amount of TUNEL positive (Fig. 2C,) and Annexin V positive cells (Figs. 2D), without stopping the cell cycle. In contrast, 20 µM DhL triggered cell cycle accumulation in the G1 phase (Figs. S2A, B and 5A, B) of cells that had lower levels of broken DNA (Fig. 3A, B, C) and that accumulated senescence markers (Fig. 7A, B, C, D). Although it is tempting to interpret the nature of this sharp concentration-dependent choice between apoptosis and senescence, the relevance of our findings relies on the capacity of DhL to display strategies against cancer cell proliferation in an additive fashion. In studies of HCT116 cells the sensitivity to DhL appears to be higher than in HeLa cells. DhL at a concentration of 10 µM was sufficient to promote p53-dependent senescence (Fig. S4B), in agreement with previous demonstrations of the role of p53 activation in cellular choices of a senescence pathway [48]. Other p53-independent signals may also contribute to the anti-proliferative effect of higher DhL concentrations (Fig. 6B lower panels, 20 µM DhL treatment; note the reduced difference between the numbers of p53-positive vs. -negative cells). DhL may therefore function in cells of differing genetic backgrounds, including p53-negative cells, triggering senescence or apoptosis in a manner that depends on both the cell type and the concentration of DhL used. This concept is relevant to the treatment of solid tumors in which access of different compounds to the tumor tissue may be limited by the three-dimensional structure of the tumor mass. If DhL is validated as a tumor-targeting agent, the treatment dose and time frame (see Fig. 7E) may be parameters that can be effectively modulated to reduce collateral damaging effects in the clinic.

### The Mechanism of Action of DhL in Cancer Cell Treatment

The mechanism of action of SLs remains poorly understood. The biological activities of these compounds have been attributed to various factors, including alkylating center reactivity, side chains, lipophilicity, and molecular or electrical features [2]. Thapsigargin, an SL compound that is currently being evaluated in phase I clinical trials, induces apoptosis via cytoplasmic signals that involve sarco/endoplasmic reticulum calcium ATPase (SERCA) pump inhibition and the release of cytochrome c from mitochondria [13]. DhL is an SL that also belongs to the guaianolide group and has the same carbon-cyclic skeleton as thapsigargin; however, we found that the molecular pathways that are activated by DhL treatment are not restricted to the cytoplasm. The increase in phosphorylated ATM, and the formation of γH2AX and 53BP1 foci strongly suggest that the signals that generated DDR markers in the nucleus may be a trigger for apoptotic and senescence programs; the latter comprise an important anticancer process that has not been previously associated with SL treatment. We believe that it is possible to chemically dissect the biological anti-proliferative activities of DhL. Although the anticancer activity of SLs of the guaianolide group has been attributed to the alpha, beta-unsaturated carbonyl group and its alkylating activity [11], we showed that a DhL with inactivated alpha-methylene lactone function (2H-DhL) affected the proliferation but not the viability of cells, suggesting that cell cycle arrest and cytotoxicity are mediated by different cellular targets of DhL [49].

Two important findings of the present study are the previously undescribed link between SLs, markers of DDR, and p53- dependent induced senescence and the fact that the degree of senescence or apoptosis induced by DhL is dose-dependent. There are clear potential advantages in the use of DhL for tumor treatment. Low doses of DhL can cause permanent cell cycle arrest in cancer cells that have damaged DNA, while higher doses can eradicate tumor cells by apoptosis.

## Supporting Information

Figure S1 **(A) The chemical structure of dehydroleucodine.** (B) Unsynchronized WI-38 and WI-38 VA cells were treated with 0, 5, 10 or 20 µM DhL for 72 h and counted every 24 h. Data are expressed as the mean ± SEM of 3 independent experiments, * p≤0.05 for WI-38 vs. WI-38 VA cells.
(TIF)

Figure S2 **20 µM DhL induces cell cycle arrest whereas 30 µM DhL induces apoptosis.** Unsynchronized (A) and synchronized (B) HeLa cells were treated with 0, 20, or 30 µM DhL for 24 or 48 h. DNA content was assessed by flow cytometry. Representative DNA distributions from 1 experiment are shown. The hypodiploid picks are indicated by arrows. (C) Representative panels for Anexin V positive HeLa cells (bright cells) treated with 0, 20, or 30 µM DhL for 48 or 72 h.
(TIF)

Figure S3 **DNA damage might result from DhL treatment.** (A) Unsynchronized HeLa cells were treated with 20 µM DhL for the indicated time points or espouse to UV radiation by 4 h, and the levels of p-ATM accumulation were assayed by immunoblot. β-actin was employed as a loading control. (B and C) Unsynchronized HeLa cells were treated with 0, 20, or 30 µM DhL for 48 h. Samples were stained with DAPI to visualize the nuclei and specific antibodies for γH2AX (B) and 53BP1 (C) were used. Representative fields are shown. Insets are magnifications of the areas indicated by boxes in the top row. Bar: 10 µm. The images shown are representative of 3 independent experiments.
(TIF)

Figure S4 **DhL-induced senescence is higher in p53+/+ than in p53−/− cells.** (A) HCT116 p53+/+ and p53−/− cells treated with 20 or 30 µM DhL were lysed at the indicated time points and used to determine p53 and p21 levels by immunoblot. The immunoblots shown are representative of 3 independent experiments with similar result. (B) HCT116 p53+/+ and p53−/− cells treated with 10 µM DhL for 48 h were used to determine SA-β-Gal activity at pH 6 *in situ*. Left: cells stained for SA-β-Gal and examined by bright field microscopy. Bar: 50 µm. Right: percentages SA-β-Gal-positive cells. Data represent the mean ± SEM of 2 experiments. * p≤0.05, ** p≤0.01, *** p≤0.001 vs. control group (0 µM). ^##^ p≤0.01 for HCT116 p53+/+ vs. HCT116 p53−/− cells.
(TIF)
