# Supplementary figures and images for: The Sesquiterpene Lactone Dehydroleucodine Triggers Senescence and Apoptosis in Association with Accumulation of DNA Damage Markers
Source: PLoS One. 2013 Jan 14;8(1):e53168. doi: 10.1371/journal.pone.0053168 (PMC3544853; doi:10.1371/journal.pone.0053168)

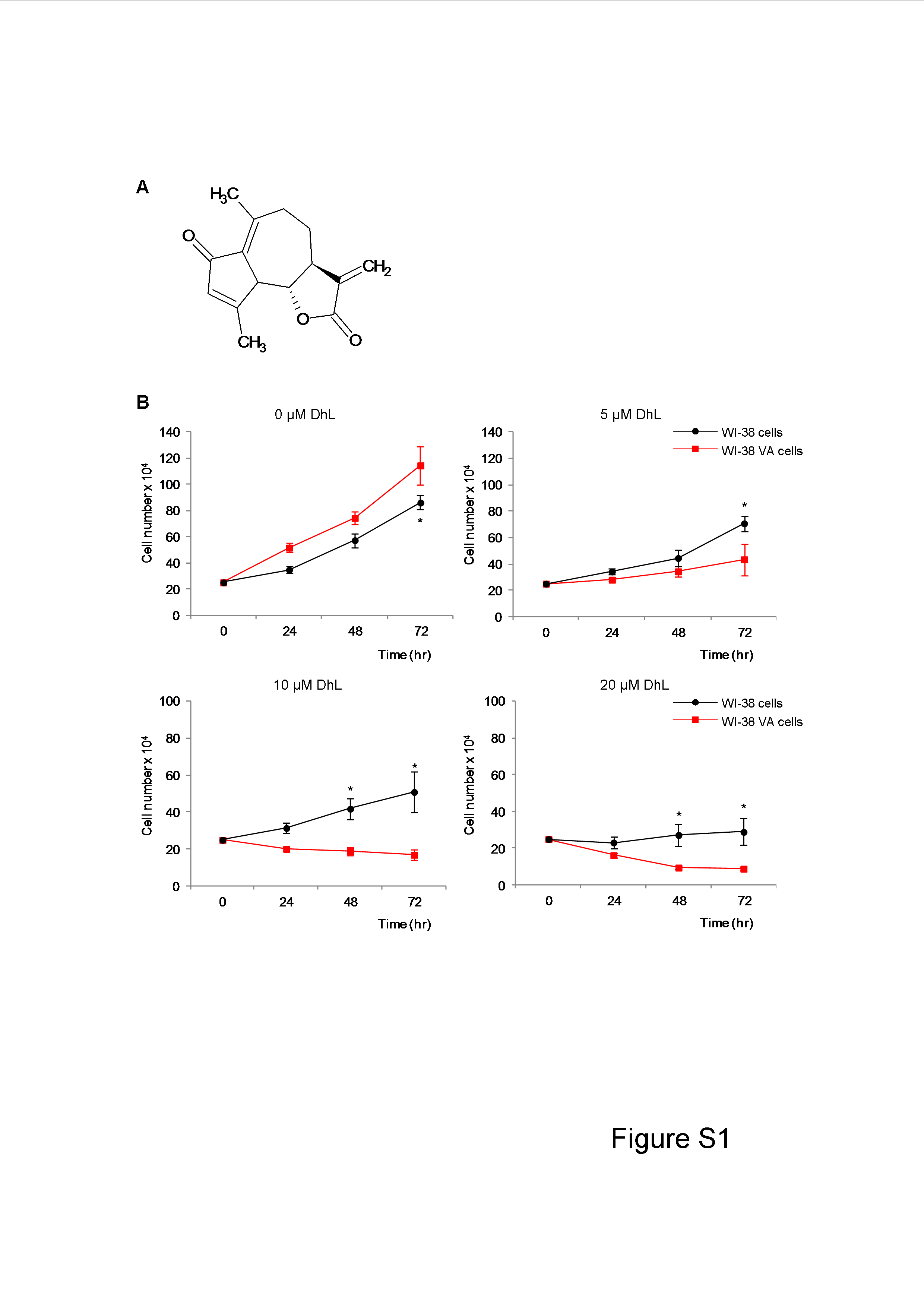

Supplement: Figure S1 — (A) The chemical structure of dehydroleucodine. (B) Unsynchronized WI-38 and WI-38 VA cells were treated with 0, 5, 10 or 20 µM DhL for 72 h and counted every 24 h. Data are expressed as the mean ± SEM of 3 independent experiments, * p≤0.05 for WI-38 vs. WI-38 VA cells. (TIF) [file pone.0053168.s001.tif]

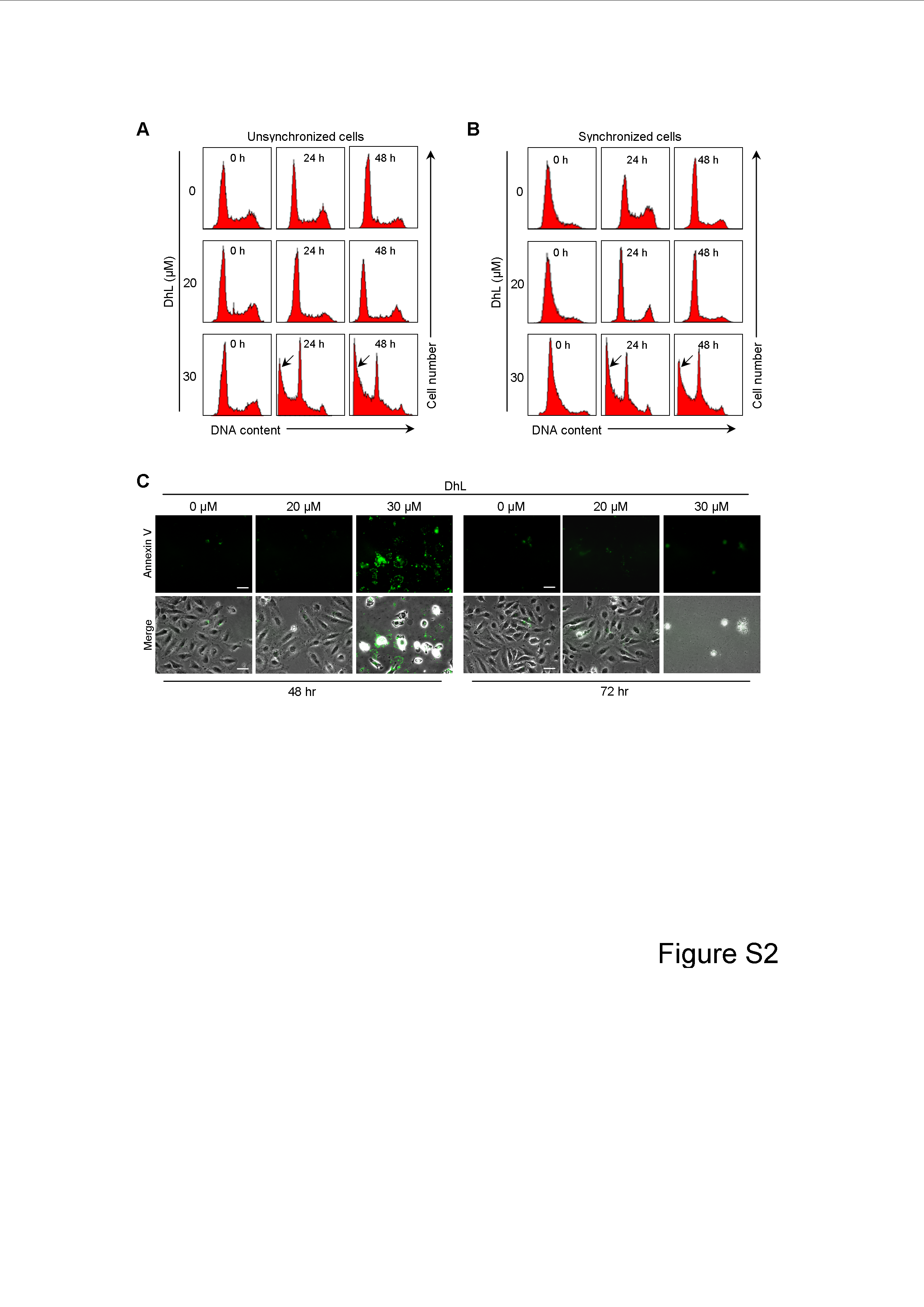

Supplement: Figure S2 — 20 µM DhL induces cell cycle arrest whereas 30 µM DhL induces apoptosis. Unsynchronized (A) and synchronized (B) HeLa cells were treated with 0, 20, or 30 µM DhL for 24 or 48 h. DNA content was assessed by flow cytometry. Representative DNA distributions from 1 experiment are shown. The hypodiploid picks are indicated by arrows. (C) Representative panels for Anexin V positive HeLa cells (bright cells) treated with 0, 20, or 30 µM DhL for 48 or 72 h. (TIF) [file pone.0053168.s002.tif]

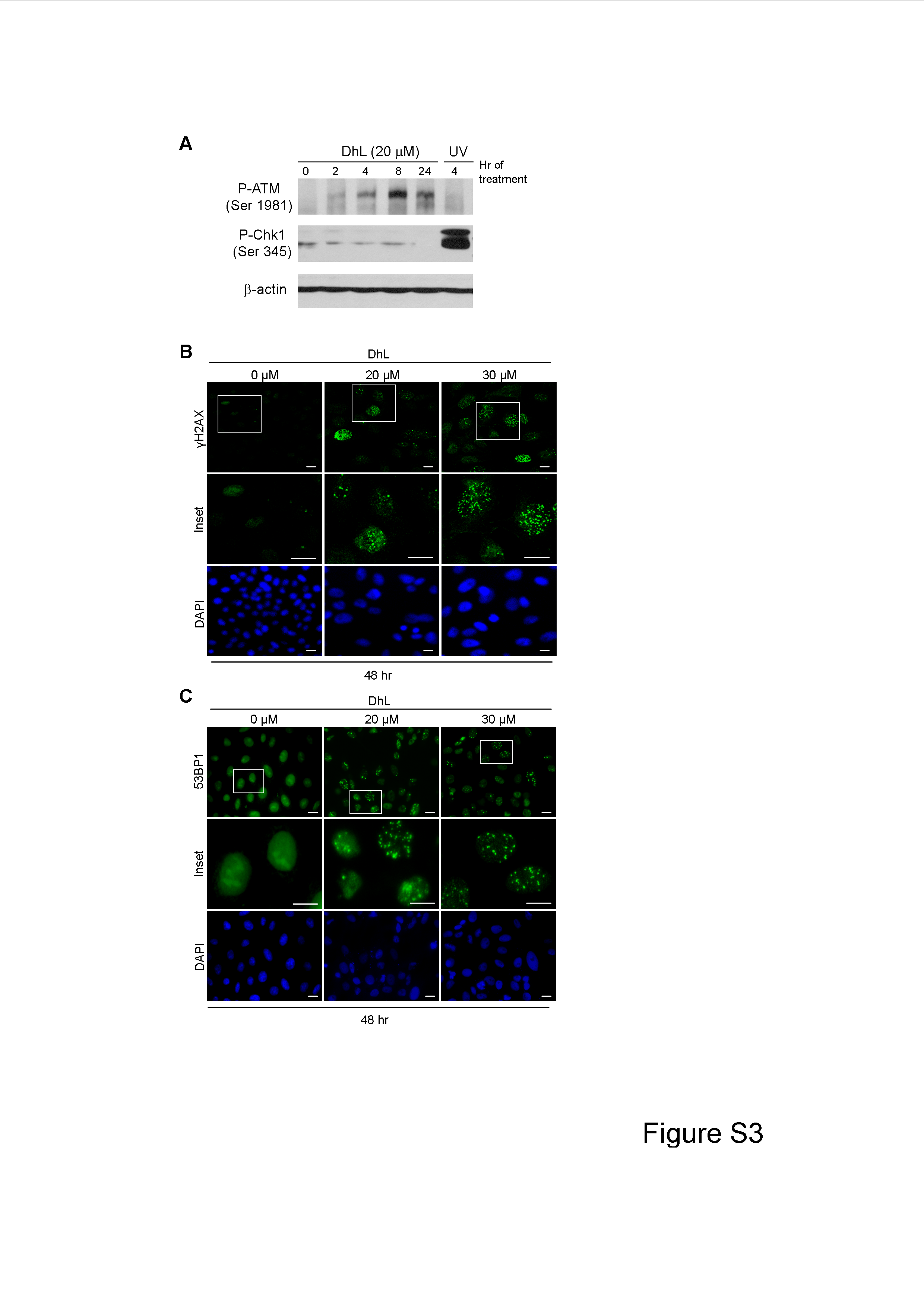

Supplement: Figure S3 — DNA damage might result from DhL treatment. (A) Unsynchronized HeLa cells were treated with 20 µM DhL for the indicated time points or espouse to UV radiation by 4 h, and the levels of p-ATM accumulation were assayed by immunoblot. β-actin was employed as a loading control. (B and C) Unsynchronized HeLa cells were treated with 0, 20, or 30 µM DhL for 48 h. Samples were stained with DAPI to visualize the nuclei and specific antibodies for γH2AX (B) and 53BP1 (C) were used. Representative fields are shown. Insets are magnifications of the areas indicated by boxes in the top row. Bar: 10 µm. The images shown are representative of 3 independent experiments. (TIF) [file pone.0053168.s003.tif]

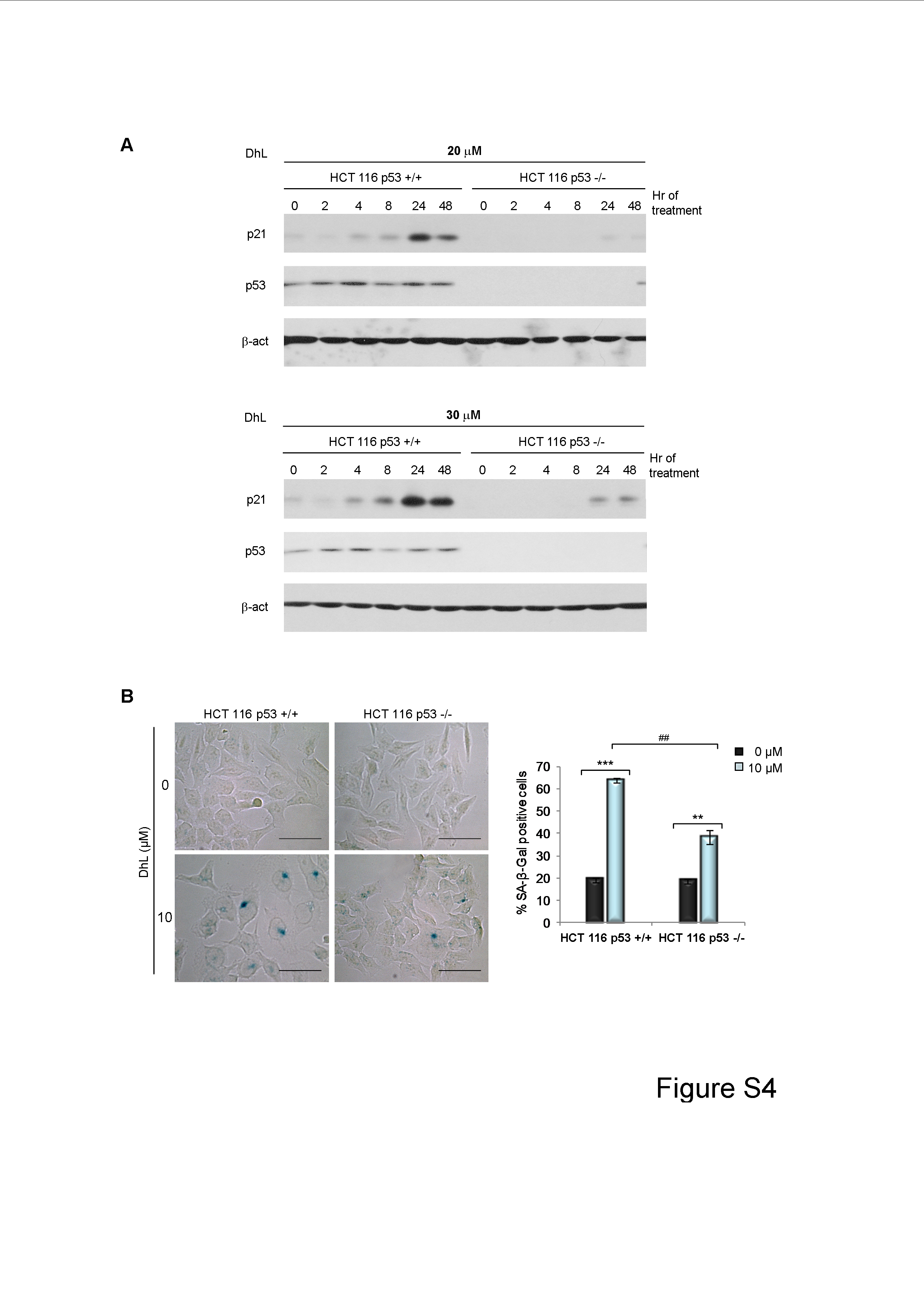

Supplement: Figure S4 — DhL-induced senescence is higher in p53+/+ than in p53−/− cells. (A) HCT116 p53+/+ and p53−/− cells treated with 20 or 30 µM DhL were lysed at the indicated time points and used to determine p53 and p21 levels by immunoblot. The immunoblots shown are representative of 3 independent experiments with similar result. (B) HCT116 p53+/+ and p53−/− cells treated with 10 µM DhL for 48 h were used to determine SA-β-Gal activity at pH 6 in situ. Left: cells stained for SA-β-Gal and examined by bright field microscopy. Bar: 50 µm. Right: percentages SA-β-Gal-positive cells. Data represent the mean ± SEM of 2 experiments. * p≤0.05, ** p≤0.01, *** p≤0.001 vs. control group (0 µM). ## p≤0.01 for HCT116 p53+/+ vs. HCT116 p53−/− cells. (TIF) [file pone.0053168.s004.tif]
